# Temporomandibular disorder in children with juvenile idiopathic arthritis with and without temporomandibular joint involvement compared to controls – a two-year prospective multicenter cohort study

**DOI:** 10.1186/s12903-026-07738-4

**Published:** 2026-01-24

**Authors:** Josefine M. Halbig, Peter Stoustrup, Kasper Dahl Kristensen, Paula Frid, Veronika Rypdal, Nils Thomas Songstad, Thomas A. Augdal, Johannes Fischer, Elisabeth G. Gil, Lena Cetrelli, Anette Lundestad, Oskar Angenete, Stein Magnus Aukland, Karin Tylleskär, Annika Rosen, Marit S. Skeie, Marite Rygg, Karen Rosendahl, Birgitta Jönsson, Ellen Nordal

**Affiliations:** 1 Public Dental Health Competence Centre of Northern Norway (TkNN), Postbox 2406, Tromsø, 9271 Norway; 2 Department of Clinical Medicine, Research Group of Child and Adolescent Health, UiT The Arctic University of Norway, Tromsø, Norway; 3 Section of Orthodontics, Department of Dentistry and Oral Health, Aarhus University, Aarhus, Denmark; 4 Department of Clinical Dentistry, UiT The Arctic University of Norway, Tromsø, Norway; 5 Department of Pediatric and Adolescent Medicine, University Hospital of North Norway, Tromsø, Norway; 6 Department of Radiology, University Hospital of North Norway, Tromsø, Norway; 7 Department of Clinical Medicine, UiT The Arctic University of Norway, Tromsø, Norway; 8 Department of Clinical Dentistry, University of Bergen, Bergen, Norway; 9 Knarvik Orthodontic Clinic, Alver, Norway; 10 Center for Oral Health Services and Research (TkMidt), Trondheim, Norway; 11 Department of Clinical and Molecular Medicine and Health Science, Norwegian University of Science and Technology (NTNU), Trondheim, Norway; 12 Department of Pediatrics, St. Olavs Hospital HF, Trondheim University Hospital, Trondheim, Norway; 13 Department of Circulation and Medical Imaging, Norwegian University of Science and Technology (NTNU), Trondheim, Norway; 14 Department of Radiology and Nuclear Medicine, St Olav Hospital HF, Trondheim University Hospital, Trondheim, Norway; 15 Department of Radiology, Haukeland University Hospital, Bergen, Norway; 16 Department of Clinical Medicine, University of Bergen, Bergen, Norway; 17 Department of Pediatric and Adolescent Medicine, Haukeland University Hospital, Bergen, Norway; 18 Department of Oral and Maxillofacial Surgery, Haukeland University Hospital, Bergen, Norway; 19 Department of Periodontology, Institute of Odontology, University of Gothenburg, Gothenburg, Sweden

**Keywords:** Juvenile arthritis, Craniomandibular disorders, Orofacial symptoms, Myalgia, Temporomandibular joint involvement

## Abstract

**Objectives:**

This study compares the prevalence of orofacial signs and symptoms of temporomandibular disorder (TMD) in children with juvenile idiopathic arthritis (JIA) and controls at two study visits two years apart. We also examine the prevalence of TMD diagnoses and their association with general disease activity in children with JIA.

**Methods:**

In the NorJIA cohort study, children with JIA aged 4 to 16 years were recruited consecutively from three pediatric rheumatology clinics in Norway and together with an age- and sex-matched non-JIA control group from public dental health service clinics. Children with JIA were classified into temporomandibular joint (TMJ) involvement and no TMJ involvement groups on the basis of magnetic resonance imaging findings. An adapted version of the Diagnostic Criteria for TMD protocol was applied to assess myalgia, arthralgia, headache attributed to TMD, and disc displacement in all children at both visits.

**Results:**

Fifty-five children with JIA and TMJ involvement, 132 with JIA without TMJ involvement, and 189 controls completed TMD examinations at both visits. At Visit I, at least one TMD diagnosis was present in 49% of the children with JIA and TMJ involvement, 19% with JIA without TMJ involvement, and 5% of the controls; after two years, these frequencies were 38%, 16%, and 7%, respectively. Children with active JIA disease at baseline had a higher risk of orofacial myalgia (OR 10.5, 95% CI 3.1–36.4).

**Conclusions:**

TMD was more common in children with JIA than in non-JIA controls. Active JIA disease increased the risk of temporomandibular myalgia, regardless of TMJ involvement. Pediatric rheumatologists and dentists should regularly monitor the orofacial region in all children with JIA.

**Trial registration:**

Registered retrospectively on clinicaltrials.gov (NCT03904459, 03/04/2019).

**Supplementary Information:**

The online version contains supplementary material available at 10.1186/s12903-026-07738-4.

## Background

Juvenile idiopathic arthritis (JIA) is the most common rheumatic disease in children. It is a heterogeneous entity with seven different categories defined as systemic arthritis, oligoarthritis, rheumatoid factor (RF)-positive and RF-negative polyarthritis, psoriatic arthritis, enthesitis-related arthritis, and undifferentiated arthritis [1]. The treatment of JIA is multifaceted, and multidisciplinary cooperation is crucial. Early aggressive drug treatment with a treat-to-target approach is increasingly recommended [2]. The disease is often not manageable with non-steroidal anti-inflammatory drugs (NSAIDs) and/or local intra-articular corticosteroid injections (IACS) alone, and synthetic and/or biologic disease-modifying antirheumatic drugs (DMARDs) are necessary [2]. The temporomandibular joint (TMJ) can be affected by arthritis, just as any other joint [3–5]. TMJ arthritis may impact the whole temporomandibular complex, leading to joint and muscle symptoms, dentofacial deformities, malocclusions, and reduced quality of life [4–7].

Temporomandibular disorder (TMD) is an umbrella term encompassing three groups of clinical conditions: 1) TMJ disorders, 2) masticatory muscle disorders, and 3) headaches [8, 9]. While some TMD diagnoses are based on patient history (symptoms) and clinical examination (clinical signs), imaging is mandatory for diagnosing disc displacement, arthritis, and arthrosis [8]. Several protocols have been developed to examine the temporomandibular complex [10–13]. Studies investigating dysfunction in the temporomandibular region in children and adolescents with JIA have utilized either the Helkimo index [14], the World Health Organization tool [15], the Craniomandibular Index [16], the Diagnostic Criteria for Temporomandibular Disorders (DC/TMD) protocol [17–20], or their own protocols [21–23].

Interdisciplinary consensus-based recommendations for clinical orofacial examinations in patients with JIA were published in 2017 by the European TMJ Research Network, now referred to as TMJaw [24]. According to the recommendations, the location, intensity, and frequency of orofacial symptoms reported by the patient or a proxy should be recorded, together with the type of situation or activity that elicits the symptom. The orofacial examination in children with JIA by pediatric rheumatologists and dentists should include palpation of masticatory muscles and the TMJ, as well as the assessment of TMJ sounds and pain during mandibular movement. Additionally, the mandibular function, as well as facial morphology and symmetry, should be evaluated [24].

Studies investigating the temporomandibular complex in children and adolescents with JIA are often small and frequently lack reports on control groups [17, 21, 22, 25, 26]. The use of various protocols for clinical examination makes it challenging to compare results or combine data to analyze the prevalence of TMD in larger samples or meta-analyses [26]. Publications on longitudinal data comparing TMD diagnoses in children and adolescents with and without JIA are limited; however, there are retrospective [27] and prospective longitudinal [28, 29] studies on TMJ involvement and orofacial symptoms in JIA. There is limited knowledge on whether children with JIA have a higher prevalence of TMD diagnoses, including myalgia, arthralgia, headache attributed to TMD, and disc displacement, regardless of verified TMJ involvement. Additionally, associations between these TMD diagnoses and JIA disease activity have not yet been established, to our knowledge.

In this study, we aim to compare the prevalence of orofacial signs and symptoms of TMD in a cohort of consecutive children with JIA, with and without TMJ involvement, and in controls at two study visits two years apart. Furthermore, we aim to examine the prevalence of TMD diagnoses in the three groups and to assess the association between TMD diagnoses and disease activity in children with JIA.

## Methods

### Study design and population

The Norwegian JIA study (www.norjia.com) is a prospective longitudinal multicenter cohort study with a comparative design, including 228 children and adolescents with JIA and an age- and sex-matched non-JIA control group. The study group and controls were recruited and enrolled between March 2015 and August 2018 (Visit I), with a recall study visit (Visit II) approximately two years later, which was completed in October 2020, using the same questionnaires and clinical examination protocols as in Visit I. Pediatric rheumatologists at the three university hospitals in Bergen, Trondheim, and Tromsø invited consecutive patients aged 4 to 16 years who were diagnosed with JIA according to the International League of Associations for Rheumatology (ILAR) criteria [1]. These hospitals are responsible for all children with JIA in Western, Middle, and Northern Norway, and all health care is free of charge for children under 16 years of age. All children and adolescents ≤ 18 years old in Norway have regular routine examinations at public dental health service clinics, free of charge. Children and adolescents without JIA, listed for routine examination at the public dental health services in Bergen (Western Norway), Stjørdal (Middel Norway), and Tromsø (North Norway), were invited by dentists to serve as a non-JIA control group, and were matched according to age and sex with the participants with JIA. The exclusion criterion for both groups was the lack of written consent to participate.

This sub-study included all NorJIA participants who underwent and completed the TMD examination at both visits. Participants with JIA who did not undergo magnetic resonance imaging (MRI) at Visit I were excluded from this sub-study.

### Orofacial symptoms

Before the clinical TMD examination, an interview was conducted with the child and their parent. Participants were asked if they had ever experienced orofacial pain and whether they had experienced it within the last 30 days. Participants who reported pain during the last 30 days were asked additional questions according to the validated Temporomandibular Joint Arthritis Working Group (TMJaw) recommendations for assessing orofacial symptoms in JIA [24, 30], as presented in Supplemental, Table S1. The symptoms of *pain when chewing*, *pain when opening the mouth wide,* and *pain when talking for a long time* were combined into a single variable referred to as “*Pain on function”*.

### Clinical TMD assessment

The TMD assessment was part of the clinical oral examination conducted by experienced and calibrated dentists, as previously described [18]. The examination procedure was an adapted short version of the DC/TMD Axis I protocol [12], including measurements of mandibular movement, palpation of jaw muscles and the lateral pole of the TMJ, and assessment of TMJ sounds. Mandibular asymmetry, facial morphology, and mandibular deviation at maximum mouth-opening were recorded. We applied the DC/TMD definitions by Schiffman et al. [12] with adaptations [31, 32], to register the TMD diagnoses of myalgia, arthralgia, headache attributed to TMD, and disc displacement (Supplemental, Table S2). We defined limited mouth-opening as the maximal unassisted mouth-opening, including the vertical incisal overbite, measured at ≤ 32 mm for children under 10 years of age and < 40 mm for adolescents aged 10 years and older [31]. Since MRI was not available for the control group, disc displacement was determined based on clinical examination for both the JIA and control groups.

### Characteristics of the JIA cohort

Information about clinical characteristics, including age at disease onset, JIA disease duration, JIA category according to the ILAR criteria [1], the number of active joints, the cumulative joint count, and current and previous use of disease-modifying antirheumatic drugs, was collected at both visits. Additionally, the pediatric rheumatologists rated the global disease activity on a visual analog scale (VAS) from 0–10 (MDgloVAS: 0 = “no activity”, 10 = “high activity”). Proxies (for children aged < 9 years) or the child/adolescent themselves (aged ≥ 9 years) reported functional ability using the Childhood Health Assessment Questionnaire (CHAQ) [33], the disease impact on overall well-being (PRgloVAS), and pain during the last week on a 21-numbered circle VAS (range 0–10, 0 = ”no impact”/”no pain” and 10 = “most severe impact”/“most severe pain”). MDgloVAS, PRgloVAS, and global pain VAS were dichotomized as 0 and > 0. The clinical juvenile arthritis disease activity score (cJADAS 10) was calculated based on the MDgloVAS (0–10), the PRgloVAS (0–10), and the number of active joints up to the maximum count of 10 (range 0–10) [34, 35]. The cJADAS10 score was recoded to 0 = inactive disease and 1 = active disease, where active disease encompasses minimal, moderate, and high disease activity. Active disease was defined as cJADAS10 values > 1.1 for oligoarthritis (persistent and extended) and > 2.5 for polyarthritis (including systemic arthritis, polyarthritis, psoriatic arthritis, enthesitis-related arthritis, and undifferentiated arthritis) [36, 37].

### Temporomandibular joint involvement

**Fig. 1:**
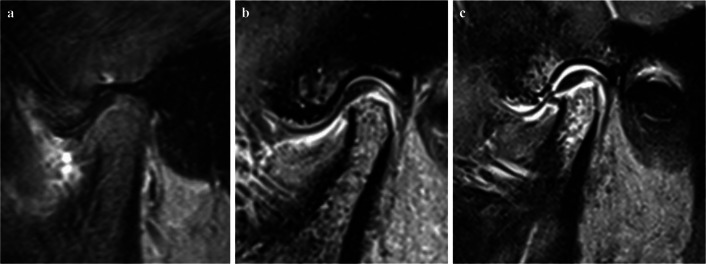
MRI assessment of TMJ involvement (sagittal oblique T1-weighted fat-saturated contrast-enhanced images), showing **a**) normal TMJ in a 12-year-old girl; **b**) mild to moderate inflammation and mild flattening of the condyle in a 16-year-old girl; and **c**) moderate to severe inflammation and a flattened condyle in a 15-year-old girl

In this study, we defined TMJ involvement as abnormalities (inflammatory and structural changes) resulting from current or previous TMJ arthritis, which is the standardized, consensus-based definition of TMJ involvement by the TMJaw Group [38]. Based on the MRI from Visit I, the children with JIA were assigned to one of the following groups: 1) with TMJ involvement or 2) without TMJ involvement, regardless of clinical signs and symptoms. All TMJ MRI examinations were performed on a 3 Tesla system (Skyra, Siemens, Erlangen, Germany), using a standardized protocol which included coronal T1-weighted, sagittal T1-weighted MPRAGE, sagittal/oblique fat-saturated T2-weighted, sagittal/oblique fat-saturated T1-weighted, sagittal/oblique proton density-weighted with closed and open mouth and sagittal/oblique fat-saturated T1-weighted after intravenous contrast [39]. The images were interpreted by one of three experienced radiologists (OA, TAA, KR) following meticulous standardization, which included three 2-day meetings and subsequent analysis of intra- and interobserver reliability for various features in both the inflammatory and the osteochondral domains [39]. For the present paper, we used the initial radiology reports, to group children into either “TMJ involvement” (Fig. 1b and c: inflammation and/or structural change) or “No TMJ involvement” (Fig. 1a: no definite inflammation or structural change, including equivocal findings, that might represent normal variation, such as minimal irregularities of the articular surface, tiny foci of bone marrow edema-like change, or asymmetrical distribution/small amounts of joint fluid) [39].

### Treatment during the study period

In this observational longitudinal study, all participants with JIA followed local treatment guidelines based on the individual decisions of their treating physician. The treatment included systemic therapies such as DMARDs, NSAIDs, physical therapy, and/or local steroid injections. A total of 12 participants with JIA received a TMJ glucocorticoid injection: 11 in the TMJ involvement group shortly after Visit I, and one participant with JIA without TMJ involvement at Visit I developed TMJ arthritis six months later and was treated with a TMJ glucocorticoid injection. Participants in both the JIA and control groups with TMD diagnoses, such as myalgia, arthralgia, disc displacement, and/or headache attributed to TMD, were treated with one or more of the following: information and instruction in physical exercises, or occlusal splints according to the clinical guidelines.

### Statistical methods

All statistical analyses were conducted using STATA version 18 software (STATA Corp., College Station, Texas, USA). Means, medians, standard deviations (SD), interquartile ranges (IQR), and frequencies were reported for descriptive data. Chi-squared, Fisher’s exact, Kruskal–Wallis, one-way ANOVA with Bonferroni correction, and Mann–Whitney U tests were applied to evaluate differences between groups. McNemar’s test was used to assess changes within each group over time. When comparing three groups (A = JIA with TMJ involvement, B = JIA without TMJ involvement, and C = non-JIA controls), we first performed a primary analysis for the three groups together (A, B, C) and subsequently conducted pairwise secondary analysis for primary significant results (A vs. B, B vs. C, A vs. C). To correct for multiple analyses, we applied a false discovery rate of 5% (q = 0.05) for both primary and secondary analyses [40]. Logistic regression analyses were performed to examine the associations between TMD and JIA disease activity, both unadjusted and adjusted (for age, sex, and TMJ involvement), for TMD diagnoses present in a minimum of 20 children with JIA.

## Results

### Cohort characteristics

**Fig. 2:**
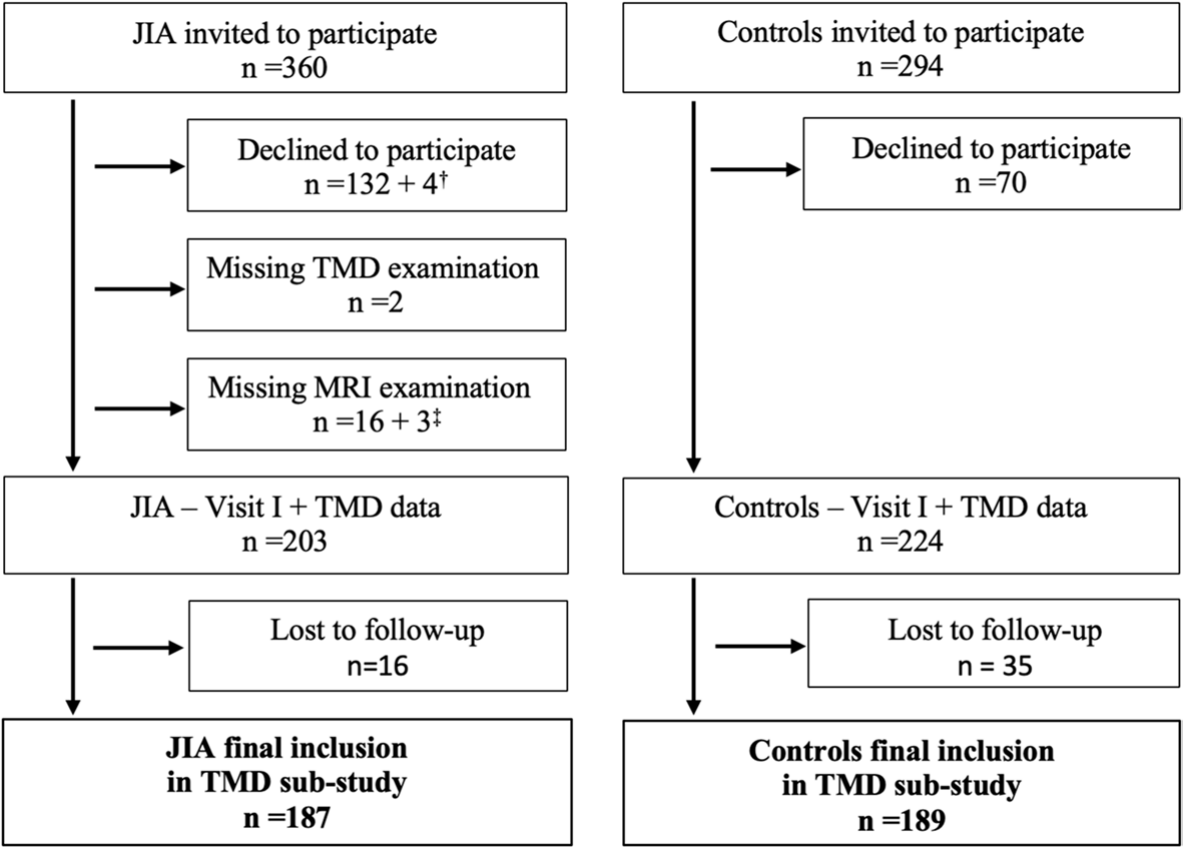
Participant flow diagram of children and adolescents with juvenile idiopathic arthritis (JIA) and controls. ^†^ Participated in the main study but declined oral examination. ^‡^ Poor MRI quality not possible to assess TMJ involvement

A total of 187 children with JIA who underwent an MRI examination at Visit I and 189 non-JIA controls completed the TMD examinations at both study visits (Fig. 2). According to the MRI assessment at Visit I, TMJ involvement was present in 55 (29%) children with JIA (Table 1). The group of children with JIA and TMJ involvement was older, had a longer JIA disease duration, and included more females compared to the group of children with JIA without TMJ involvement. Additionally, a greater proportion of children with JIA and TMJ involvement had a polyarticular disease course, active disease, and functional limitations at Visit I (Table 1). The mouth-opening capacity was significantly lower in children with JIA and TMJ involvement (Mean ± SD: 46.9 ± 6.9 mm) compared to children without TMJ involvement (Mean ± SD: 51.1 ± 7.7 mm, *p* < *0.001*) at Visit I (Table 1). However, there were no significant differences in mouth-opening capacity between children with JIA without TMJ involvement and controls (Mean ± SD: 52.2 ± 7.4 mm, *p* = *0.16*) at Visit I.

**Table 1:** Visit I-Distribution of characteristics among children with juvenile idiopathic arthritis (*N =* 187) and controls (*N =* 189)

**Variable**	**A—JIA with** **TMJ involvement**^**†**^		**B—JIA without** **TMJ involvement**^**†**^		**C—Controls**^**†**^		***p*-value**^‡^
Female gender	76	42/55	52	69/132	61	115/189	***0.01*** **< *0.01***^*(A, B)*^ *0.13*^*(B, C)*^ *0.03*^*(A, C)*^
Age at visit, years (median, IQR)	13.9	11.4–15.2	12.4	9.5–14.5	12.4	9.6–14.8	***0.04*** ***0.02***^*(A, B)*^* 0.82*^*(B, C)*^ ***0.02***^*(A, C)*^
Mouth-opening capacity^§^, mm (mean, SD)	46.9	6.9	51.1	7.7	52.2	7.4	**< *0.001*** **< *0.001***^*(A, B)*^* 0.16*^*(B, C)*^ < ***0.001***^*(A, C)*^
JIA disease characteristics							
Age at disease onset, years (median, IQR)	6.1	2.1–10.7	7	2.7–10.3			*0.62*
Disease duration, years (median, IQR)	5.8	3.4–10.3	4	2.4–7.7			*0.02*
Polyarticular disease course (> 4 affected joints)	76	42/55	40	54/132			**< *0.001***
Oligoarticular JIA category^¶^	33	18/55	50	62/132			*0.07*
JIA category							
Systemic JIA	2	1/55	4	5/132			
Oligoarthritis persistent	16	9/55	39	52/132			
Oligoarthritis extended	16	9/55	8	10/132			
Polyarthritis RF-	36	20/55	17	23/132			
Polyarthritis RF +	2	1/55	1.5	2/132			
Psoriatric JIA	5	3/55	3	4/132			
Enthesitis relatet JIA	13	7/55	11	15/132			
Undifferentiated JIA	9	5/55	16	21/132			
DMARDs ongoing	78	43/55	64	84/132			*0.05*
sDMARDs only	27	15/55	29	38/132			*0.83*
bDMARDs with/without sDMARDs	51	28/55	35	46/132			*0.04*
Active joints > *0*	42	23/55	17	23/132			**< *0.001***
ESR ≥ 20 mm/h	7	4/55	3	4/130			*0.24*
MDglobalVAS > 0	58	32/55	29	37/132			**< *0.001***
PRglobalVAS > 0	83	44/53	70	90/129			*0.07*
Global pain VAS > 0	70	37/53	60	77/129			*0.20*
CHAQ > 0	70	37/53	52	67/128			*0.03*
cJADAS10 (median, IQR)	3	1–6.5	1.5	0–4.5			**< *0.01***
cJADAS10 > 1.1/2.5^ǁ^	64	34/53	46	59/129			*0.02*

*Abbreviations*: *JIA* Juvenile idiopathic arthritis, *TMJ* Temporomandibular joint, *IQR* Interquartile range, *mm* millimeter, *SD* Standard deviation, *s/b*DMARDs synthetic/biologic disease-modifying antirheumatic drugs, *ESR* Erythrocyte sedimentation rate, *MDglobalVAS* Physician's global assessment of disease activity, *VAS* Visual analog scale, *PRglobalVAS* Parent's global assessment of disease activity, *CHAQ* Childhood Health Assessment Questionnaire, *cJADAS10* clinical juvenile arthritis disease activity score, with a maximum active joint count of 10

^†^Data are shown as percentages and n/N, number observed/total number assessed, excluding missing values, if not otherwise specified

^‡^Chi-squared-, Kruskal–Wallis-, One-way ANOVA (Bonferroni), Mann–Whitney U-, T-, and Fischer’s exact-test; p-values < 0.05 are marked in bold if false discovery rate q-values < 0.05

^§^Mouth-opening capacity = non-assisted mouth-opening capacity + vertical overbite

^¶^*Oligoarticular* (including persistent and extended) *versus non-oligoarticular* (including systemic onset, polyarthritis RF negative, polyarthritis RF positive, psoriatic JIA, enthesitis-related arthritis, and undifferentiated)

^ǁ^Active disease, including minimal, moderate, and high disease activity if cJADAS10 > 1.1 for *oligoarticular* and > 2.5 for *non-oligoarticular JIA*

### Distribution of orofacial signs and symptoms

At Visit I, 29% (*n =* 16) of children with JIA and TMJ involvement reported TMJ pain, compared to 12% (*n =* 16) of those with JIA without TMJ involvement, and 3% (*n =* 5) of the controls (Table 2). There was no significant change in the frequency of reported TMJ pain and orofacial symptoms at Visit II compared to Visit I in any of the groups. At both visits, the highest frequency of orofacial symptoms was reported by children with JIA and TMJ involvement (Table 2). TMJ and muscle pain were also reported significantly more often by children with JIA without TMJ involvement compared to controls at both visits (Table 2). Across all three groups, there was a significant decrease in the number of children reporting muscular pain on palpation at Visit II compared to Visit I (Table 2). Pathologically reduced mouth-opening capacity (≤ 32 mm for children under 10 years of age and < 40 mm for adolescents aged 10 years and older) was present in significantly more children with JIA and TMJ involvement compared to children with JIA without TMJ involvement (Visit II) and to controls (Visits I and II, Table 2). Only two participants who presented with reduced mouth-opening capacity at Visit I still had reduced mouth-opening capacity at Visit II. Three participants with TMJ involvement and reduced mouth-opening capacity received a corticosteroid injection during the study period. For these participants, reduced mouth-opening capacity was no longer observed at Visit II. The other nine participants who received an injection during the study period had neither reduced mouth-opening capacity at Visit I nor at Visit II (Data not shown).

**Table 2:** Symptoms and clinical signs of temporomandibular disorder among children with juvenile idiopathic arthritis and controls

	**A** **JIA with** **TMJ involvement** *N =* 55	**B** **JIA without** **TMJ involvement** *N =* 132	**C** **Controls** *N =* 189	**p-value**^**†**^			
				Primary analysis	Secondary analysis		
	% (n)	% (n)	% (n)		A vs B	B vs C	A vs C
**Symptoms**							
TMJ pain							
Visit I	29 (16)	12 (16)	3 (5)	**< *0.001***	***0.01***	**< *0.01***	**< *0.001***
Visit II	31 (17)	13 (17)	5 (9)	**< *0.001***	**< *0.01***	***0.01***	**< *0.001***
Muscle pain							
Visit I	40 (22)	16 (21)	4 (8)	**< *0.001***	**< *0.001***	**< *0.001***	**< *0.001***
Visit II	33 (18)	15 (20)	4 (8)	**< *0.001***	***0.01***	**< *0.01***	**< *0.001***
Pain on function							
Visit I	38 (21)	10 (13)	2 (3)	**< *0.001***	**< *0.001***	***0.001***	**< *0.001***
Visit II	24 (13)	11 (15)	4 (7)	**< *0.001***	***0.03***	***0.01***	**< *0.001***
Chewing limitations							
Visit I	16 (9)	2 (3)	0.5 (1)	**< *0.001***	**< *0.01***	*0.31*	**< *0.001***
Visit II	13 (7)	2 (3)	2 (3)	**< *0.01***	***0.01***	*0.69*	**< *0.01***
Morning stiffness in the jaw							
Visit I	18 (10)	5 (6)	0.5 (1)	**< *0.001***	**< *0.01***	***0.02***	**< *0.001***
Visit II	16.4 (9)	5 (6)	2 (4)	**< *0.01***	***0.01***	*0.33*	**< *0.001***
Jaw getting stuck							
Visit I	11 (6)	5 (6)	2 (3)	***0.01***	*0.10*	*0.17*	***0.01***
Visit II	11 (6)	3 (4)	1 (2)	**< *0.01***	*0.07*	*0.23*	**< *0.01***
**Clinical Signs**							
TMJ pain on palpation							
Visit I	55 (30)	35 (46)	21 (40)	**< *0.001***	***0.01***	***0.01***	**< *0.001***
Visit II	47 (26)	26.5 (35)	12 (22)**	**< *0.001***	***0.01***	**< *0.01***	**< *0.001***
Muscular pain on palpation							
Visit I	60 (33)	36 (47)	22 (41)	**< *0.001***	**< *0.01***	***0.01***	**< *0.001***
Visit II	40 (22)**	20.5 (27)***	14 (26)*	**< *0.001***	***0.01***	*0.11*	**< *0.001***
M. temporalis pain on palpation							
Visit I	38 (21)	22 (29)	12 (23)	**< *0.001***	***0.02***	***0.02***	**< *0.001***
Visit II	25.5 (14)	11 (14)**	9.5 (18)	***0.01***	***0.01***	*0.75*	**< *0.01***
M. masseter pain on palpation							
Visit I	60 (33)	30 (39)	16 (31)	**< *0.001***	**< *0.001***	***0.005***	**< *0.001***
Visit II	36 (20)***	16 (21)***	8.5 (16)*	**< *0.001***	**< *0.01***	*0.04*	**< *0.001***
Limited mouth-opening^‡^							
Visit I	9 (5)	3 (4)	2 (3)	***0.03***	*0.13*	*0.45*	***0.02***
Visit II	13 (7)	1 (1)	2 (4)	**< *0.01***	**< *0.01***	*0.65*	**< *0.01***

Exact McNemar-test (differences in frequencies in one group between Visits I and II): * *p ≤* 0.05, ** *p ≤* 0.01, ***p ≤ 0.001

*Abbreviations*: *JIA* Juvenile idiopathic arthritis, *TMJ* Temporomandibular joint, *M* Musculus

^†^Chi-squared-/Fischer’s exact-test (differences in frequencies between groups at one time point); p-values < 0.05 are marked in bold if false discovery rate q-values < 0.05

^‡^Limited maximal unassisted mouth-opening (including vertical overbite) ≤ 32 mm for children < 10 years of age and < 40 mm for adolescents 10 years and older

### Temporomandibular disorder

At Visit I, 28% (*n =* 52) of the children with JIA presented with at least one of the TMD diagnoses — myalgia, arthralgia, headache attributed to TMD, or disc displacement — compared to 5% (*n =* 9) in the non-JIA control group. Ten percent (*n =* 18) of all children with JIA had two or more TMD diagnoses, compared to 0.5% (*n =* 1) of the controls (Supplemental, Table S3). The frequency of having at least one TMD diagnosis and having two or more TMD diagnoses was highest in children with TMJ involvement at both visits (Table 3).

**Table 3:** Prevalence of temporomandibular disorder (TMD) in children with juvenile idiopathic arthritis (JIA) and controls

	**A** **JIA with** **TMJ involvement** *N =* 55	**B** **JIA without** **TMJ involvement** *N =* 132	**C** **Controls** *N =* 189	***p*-value**^**†**^			
				Primary analysis	Secondary analysis		
	% (n)	% (n)	% (n)		A vs B	B vs C	A vs C
Visit I							
At least one TMD diagnosis^‡^	49 (27)	19 (25)	5 (9)	**< *0.001***	**< *0.001***	**< *0.001***	**< *0.001***
Two or more TMD diagnoses^‡^	20 (11)	5 (7)	0.5 (1)	**< *0.001***	**< *0.01***	***0.01***	**< *0.001***
Visit II							
At least one TMD diagnosis^‡^	38 (21)	16 (21)	7 (13)	**< *0.001***	**< *0.01***	***0.01***	**< *0.001***
Two or more TMD diagnoses^‡^	18 (10)	4 (5)	2 (3)	**< *0.001***	**< *0.01***	*0.28*	**< *0.001***

*Abbreviations*: *TMD* Temporomandibular disorder, *JIA* Juvenile idiopathic arthritis, *TMJ* Temporomandibular joint

^†^Chi-squared- and Fischer’s exact-test; *p*-values < 0.05 are marked in bold if false discovery rate q-values < 0.05

^‡^TMD diagnoses: Myalgia, arthralgia, headache attributed to TMD, or disc displacement, not including arthritis or arthrosis

**Fig. 3:**
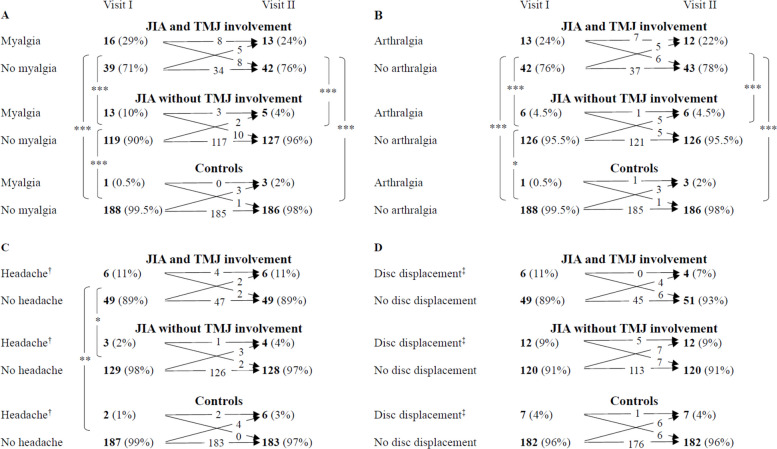
Distribution of **A** - Myalgia, **B** - Arthralgia, **C** - headache attributed to TMD, and **D** - Disc displacement according to group affiliation (JIA with TMJ involvement, JIA without TMJ involvement, and controls) at Visits I and II. TMJ involvement was defined as signs of inflammation and/or structural damage on magnetic resonance imaging from Visit I. Abbreviations: TMD, temporomandibular disorder; JIA, juvenile idiopathic arthritis; TMJ, temporomandibular joint; Fischers Exact/Chi-square as appropriate, *p*-value: * ≤ *0.05*, ** ≤ *0.01*, *** ≤ *0.001*, when false discovery rate q-values < 0.05

The distributions of myalgia, arthralgia, headache attributed to TMD, and disc displacement at Visits I and II are presented in Fig. 3. While the number of children with TMD diagnoses remained relatively stable, a substantial number of the children with a specific TMD diagnosis at Visit I did not meet the criteria at Visit II, and vice versa. At Visits I and II, significantly more children with JIA and TMJ involvement had myalgia and arthralgia compared to both children with JIA without TMJ involvement and controls (Fig. 3A and B). At Visit I, we also found significantly more children with JIA without TMJ involvement who had myalgia and arthralgia compared to controls (Fig. 3A and B). Headache attributed to TMD was significantly more prevalent in children with JIA and TMJ involvement compared to both children with JIA without TMJ involvement and controls at Visit I (Fig. 3C). For headache attributed to TMD at Visit II and disc displacement at Visits I and II, there were no significant differences between the three groups (Fig. 3C and D). According to the MRI assessment, only two children with JIA had disc displacement: one with and one without clinical signs of disc displacement.

### Associations between TMD and general JIA disease activity

The TMD diagnosis of myalgia was identified in 29 children with JIA at Visit I and qualified for further analysis of the association between the occurrence of myalgia in children with JIA and general JIA disease activity, measured by cJADAS10. At Visit I, children with JIA and active disease, had significantly higher odds of experiencing myalgia (OR: 10.5, 95% CI: 3.1–36.4, *p* <* 0.001*) compared to those with inactive disease (Supplemental, Table S4). After adjustment for TMJ involvement, sex, and age, the odds remained significantly higher (adjusted OR: 10.3, 95% CI: 2.8–37.1, *p* < *0.001*).

## Discussion

The main results of the presented study were as follows: The highest incidence of reported TMD signs and symptoms was observed in the group of children with JIA and TMJ involvement, as defined by MRI. However, even among children with JIA but without TMJ involvement, TMD signs and symptoms were significantly more common compared to non-JIA controls. The prevalence of myalgia, arthralgia, headache attributed to TMD, and disc displacement was consistent over time but with notable fluctuations in these conditions among individual participants during the two-year follow-up period. Arthralgia and myalgia were more frequent in children with JIA. Additionally, children with active JIA had higher odds of myalgia than children without active JIA.

The strengths of our study lie in its longitudinal design and the inclusion of a matched control group without JIA. The examinations were performed by calibrated examiners, who used validated questionnaires and standardized criteria to record signs and symptoms of TMD as well as the TMD diagnoses. Another strength is the use of state-of-the-art MRI machines and protocols for imaging the TMJs, including contrast-enhanced images scored by one of three experienced and meticulously calibrated radiologists.

Our results should be interpreted in light of certain limitations: The condition of disc displacement was based on patient history and clinical examination, which have shown low sensitivity [12]. Another limitation is that although the DC/TMD criteria and the questions regarding orofacial symptoms are adapted for use in children aged six years and older [32], four children with JIA and seven controls were younger than six years at the time of examination at Visit I. The questions on orofacial pain and symptoms used in our study are validated for children aged 10 years and older [30]; however, in our cohort, 24% of the children with JIA and 28% of the controls were younger than 10 years at Visit I. To address this limitation, we applied these questions during a pre-examination interview with the child and their parent to overcome the absence of validated questionnaires for young children [41]. As limited mouth-opening capacity is one of the clinical signs of TMJ arthritis [42, 43], the lack of Visit II MRI data in this sub-study further limits the discussion of the causality of limited mouth-opening capacity.

We found a significantly lower mean unassisted maximum mouth-opening capacity in children with JIA and TMJ involvement compared to children with JIA without TMJ involvement at Visit II. The low number of participants in the subgroups may explain why we did not find any significant differences in mouth-opening capacity between the groups at Visit I. In general, the mouth-opening capacity increases from childhood to adulthood, and the cutoff values we applied (≤ 32 mm for children under 10 years of age and < 40 mm for adolescents 10 years and older) are reasonable estimates of the normative values found by Stoustrup et al. [44]. It is well established that TMJ involvement in children with JIA can affect craniofacial growth, as the major growth sites of the mandible are located within the cartilage of the condyles [45]. With inadequate growth, mouth-opening capacity may not increase sufficiently throughout adolescence. Some children in the JIA TMJ involvement group may have mouth-opening limitations due to growth disturbances, but the analysis of craniofacial growth is beyond the scope of this paper.

We found a decline in the prevalence of some signs of TMD at Visit II, in contrast to Lövgren et al. [46], who reported that TMD in children and adolescents increases with age in a study conducted on a non-JIA population focusing on self-reported pain and jaw dysfunction. Rahimi et al. [29] reported the persistence of self-reported orofacial pain and functional disability in a JIA cohort at a two-year follow-up, but their participants had a mean age approximately two years older than our JIA cohort. Glerup et al. found a higher frequency of TMJ pain and chewing limitations, but a lower frequency of TMJ pain on palpation, in young adults [28]. In our cohort, children and adolescents with TMJ arthritis or other TMD diagnoses at Visit I or between the two visits were individually treated according to clinical protocols using a multidisciplinary approach. This treatment may have caused the reduction in signs of TMD, contributed to the reduction in orofacial signs and symptoms of TMD, and may have had a greater impact than the participants’ increasing age. Zwir et al. [21] reported a reduction in TMJ pain at rest and during function in a group of 75 patients with JIA at the one-year follow-up, which is in line with our results.

Our study revealed a lower prevalence of myalgia, arthralgia, headache attributed to TMD, and disc displacement in children with JIA compared to other studies [19, 25, 47]. Graue et al. [48] and Østensjø et al. [49] reported a higher prevalence of TMD and painful TMD among local Norwegian adolescent cohorts than we found in our non-JIA control group; however, the participants in their studies were older. The prevalences reported by Dimitrijevic et al. [19] and Collin et al. [17] included participants of a similar or younger age compared to our cohort, suggesting that the higher frequency of TMD in their cohort cannot be attributed to age.

We found a higher prevalence of myalgia and arthralgia in children with JIA and TMJ involvement compared to those with JIA without TMJ involvement. In contrast, Collin et al. [17] found no differences between children in these two groups; however, data on JIA disease activity were lacking, making direct comparisons difficult. The differing prevalence rates might be due to higher JIA disease activity in our study.

Our results suggest that disease activity in JIA, as indicated by a cJADAS10 score > 1.1/2.5, was strongly associated with the presence of the diagnosis myalgia during the initial clinical assessment at Visit I. Other population-based studies addressing adolescents have shown a positive association between painful TMD and pain in other parts of the body [50, 51]. The global pain VAS is not a component of the JADAS score; however, in general, pain may affect the global assessment of disease activity by physicians, and the global assessment of disease impact on overall well-being by patients or proxies, all of which are components of the JADAS. Although not directly comparable, our results support the findings of Leksell et al. [23], who reported a positive association between the number of painful chewing muscle sites and the patient’s assessment of disease activity, as well as a higher CHAQ score.

## Conclusions

Orofacial symptoms, clinical signs of TMD, and the TMD diagnoses of myalgia and arthralgia were more frequent in children with JIA, regardless of whether there was TMJ involvement, compared to non-JIA controls. Having active JIA increased the risk of experiencing temporomandibular myalgia. Since the prevalence of orofacial symptoms and TMD diagnoses showed notable fluctuations among individuals during follow-up, we suggest that dentists and pediatric rheumatologists monitor the orofacial region of children with JIA regularly and systematically, regardless of the child’s TMJ status. When clinical findings indicate TMJ involvement, an MRI of the TMJs should be considered to facilitate adequate multidisciplinary treatment strategies.

## Supplementary Information

Supplementary Material 1: Table S1. Overview of the items on orofacial symptoms as described by Stoustrup et al. [30]. Table S2. Criteria for temporomandibular disorder (TMD) conditions reproduced from Schiffman et al. [12] and adapted. Table S3. Prevalence of temporomandibular disorder (TMD) in children with juvenile idiopathic arthritis (JIA) and controls. Table S4. Active disease (cJADAS10 >1.1/2.5) in relation to myalgia in children with juvenile idiopathic arthritis (JIA).

## Data Availability

The datasets used and/or analyzed during the current study are available from the corresponding author upon reasonable request.

## Abbreviations

CHAQ: Childhood Health Assessment Questionnaire
CI: Confidence interval
cJADAS: Clinical juvenile arthritis disease activity score
DC: Diagnostic criteria
ESR: Erythrocyte sedimentation rate
ILAR: International League of Associations for Rheumatology
IQR: Interquartile range
JIA: Juvenile idiopathic arthritis
MDgloVAS: The pediatric rheumatologist’s global assessment of disease activity
MRI: Magnetic resonance imaging
NorJIA: Norwegian JIA Study
OR: Odds ratio
PRgloVAS: Patients/parents global rating of the disease’s influence on wellbeing
SD: Standard deviation
TMD: Temporomandibular disorder
TMJ: Temporomandibular joint
TMJaw group: Temporomandibular Joint Arthritis Working Group
VAS: Visual analog scale
