# Supplementary material for: Temporomandibular disorder in children with juvenile idiopathic arthritis with and without temporomandibular joint involvement compared to controls – a two-year prospective multicenter cohort study
Source: BMC Oral Health. 2026 Jan 24;26:334. doi: 10.1186/s12903-026-07738-4 (PMC12910768; doi:10.1186/s12903-026-07738-4)
Supplement: Supplementary file 1 — Supplementary Material 1: Table S1. Overview of the items on orofacial symptoms as described by Stoustrup et al. [30]. Table S2. Criteria for temporomandibular disorder (TMD) conditions reproduced from Schiffman et al. [12] and adapted. Table S3. Prevalence of temporomandibular disorder (TMD) in children with juvenile idiopathic arthritis (JIA) and controls. Table S4. Active disease (cJADAS10 >1.1/2.5) in relation to myalgia in children with juvenile idiopathic arthritis (JIA). [file 12903_2026_7738_MOESM1_ESM.pdf]

Supplemental

Table S1. Overview of the items on orofacial symptoms as described by Stoustrup et al. 2022.

Questionnaire items	Assessment for outcome
Orofacial pain ever	0) No<br>1) Yes
Orofacial pain the last 30 days	0) No<br>1) Pain is coming and going<br>2) Pain is there all the time
Pain frequency	Five ordinal outcomes:<br>0) Never<br>1) Less than once a week<br>2) Several times a week<br>3) Several times a day<br>4) All the time
Pain intensity	VAS 0-100 mm (0=no pain, 100=worst possible pain)
Pain location	Patient identification of pain locations on face map: [right and/or left side]<br>1) TMJ pain<br>2) M. masseter pain<br>3) M. temporalis pain<br>4) Pain in the cheek area
Presence of specific symptoms†	Seven questions (dichotomous outcomes "yes"/"no"):<br>1) "I felt pain when I chewed"<br>2) "I avoided hard or chewy foods because it hurt my face or jaw" = chewing limitations<br>3) "I felt pain when I opened my mouth wide (e.g. yawning)"<br>4) "I felt stiffness in my jaw muscles in the morning"<br>5) "I felt that my jaw got stuck in the open or closed position"<br>6) "I felt pain in my jaw when I talked for a long time"
Headache in the temple area the last 30 days	0) No<br>1) Yes
Joint sounds the last 30 days	0) No<br>1) Yes [right and/or left sides]

Abbreviations: VAS, visual analog scale.
†Symptoms 1, 3, and 6 were merged to "TMJ pain on function"

Ref: Stoustrup P, Rahimi H, Twilt M, et al. Assessment of Orofacial Symptoms in Juvenile Idiopathic Arthritis: Validation of a Consensus-Based Short Patient Questionnaire. J Rheumatol. Dec 2022;doi:10.3899/jrheum.220667

Table S2. Criteria for temporomandibular disorder (TMD) conditions reproduced from Schiffman et al. 2014 and adapted.

	**Myalgia**
History	1.) Pain in the temporalis and/or masseter region the last 30 days; AND<br>2.) Pain modified with jaw movement, function, or parafunction[†]
AND	
Examination	1.) Confirmation of the pain location(s) in the temporalis and/or masseter muscle; AND<br>2.) Report of familiar pain in the temporalis and/or masseter muscle(s) with at least one of the following provocation tests:<br>a. Palpation of the temporalis and/or masseter muscle(s); OR<br>b. Maximum unassisted or assisted opening movements
	**Arthralgia**
History	1.) Pain in the temporomandibular joint (TMJ) region the last 30 days; AND<br>2.) Pain modified with jaw movement, function, or parafunction[‡]
AND	
Examination	1.) Confirmation of pain location in the area of the TMJ(s); AND<br>2.) Report of familiar pain in the TMJ(s) with at least one of the following provocation tests:<br>a. Palpation of the lateral pole or around the lateral pole; OR<br>b. Maximum unassisted or assisted opening, right or left lateral, or protrusive movement(s).
	**TMD Headache**
History	1.) Headache in the temple region the last 30 days; AND<br>2.) Headache modified with jaw movement, function, or parafunction[§]
AND	
Examination	1.) Confirmation of headache location in the area of the temporalis muscle(s); AND<br>2.) Report of familiar headache in the temple area with at least one of the following provocation tests:<br>a. Palpation of the temporalis muscle(s); OR<br>b. Maximum unassisted or assisted opening, right or left lateral, or protrusive movement(s)
	**Disc displacement with reduction**
History	1.) Any TMJ noise(s) present with jaw movement or function the last 30 days; OR<br>2.) Patient report of any TMJ noise present during the exam.
AND	
Examination	Positive for at least one of the following:<br>1.) Clicking, popping, and/or snapping noise during both opening and closing movements, detected with palpation during jaw opening and closing; OR<br>2.) Positive for both of the following:<br>a. Clicking, popping, and/or snapping noise detected with palpation during opening or closing movements; AND<br>b. Clicking, popping, and/or snapping noise detected with palpation during right or left lateral, or protrusive movement(s)

Abbreviations: TMD, temporomandibular disorder
[†]Information missing for nine participants with myalgia at Visit I and four participants at Visit II,
[‡]Information missing for one participant with arthralgia at Visit I,
[§] Information missing for one participant with TMD headache at Visit I and Visit II

Ref: Schiffman E, Ohrbach R, Truelove E, et al. Diagnostic Criteria for Temporomandibular Disorders (DC/TMD) for Clinical and Research Applications. J Oral Facial Pain Headache. Winter 2014;28(1):6-27. doi:10.11607/jop.1151

Table S3. Prevalence of temporomandibular disorder (TMD) in children with juvenile idiopathic arthritis (JIA) and Controls

	**JIA** N=187	**Controls** N=189	**p-value**†
	% (n)	% (n)	
Visit I			
At least one TMD diagnosis‡	28 (52)	5 (9)	***<0.001***
Two or more TMD diagnoses‡	10 (18)	0.5 (1)	***<0.001***
Visit II			
At least one TMD diagnosis‡	22.5 (42)	7 (13)	***<0.001***
Two or more TMD diagnoses‡	8 (15)	2 (3)	***<0.01***

Abbreviations: TMD, temporomandibular disorder; JIA, juvenile idiopathic arthritis.
†Chi-squared-/Fischer's exact-test as appropriate, p-values <0.05 are marked in bold.
‡TMD diagnosis: myalgia, arthralgia, headache attributed to TMD or disc displacement; not including arthritis or arthrosis

Table S4. Active disease (cJADAS10 > 1.1/2.5) in relation to TMD diagnosis myalgia in children with juvenile idiopathic arthritis (JIA)

	**Myalgia**						
	Unadjusted regression				Adjusted regression*		
	n	OR	95%CI	*p-value*	OR	95%CI	*p-value*
Visit I, JADAS 71 ≥1							
No	89						
Yes	93	10.5	3.1-36.4	***<0.001***	10.3	2.8-37.1	**<0.001**

Abbreviations: JIA, juvenile idiopathic arthritis; JADAS, juvenile arthritis disease activity score; OR, Odds ratio; CI, Confidence interval.
Logistic regression analysis *adjusted for TMJ involvement, sex, and age.
